# Evaluating the predictions of an interoceptive inference model of bulimia nervosa

**DOI:** 10.1186/s40337-024-01010-2

**Published:** 2024-05-13

**Authors:** Maia A. Chester, Thalia Viranda, Walter H. Kaye, Laura A. Berner

**Affiliations:** 1 Department of Psychiatry, Icahn School of Medicine at Mount Sinai, New York, NY USA; 2 Department of Information Science, Cornell Tech, New York, NY USA; 3 Department of Psychiatry, University of California, San Diego, San Diego, CA USA

**Keywords:** Bayesian predictive processing, Body trust, Bulimia nervosa, Eating expectancies, Emotion regulation, Interoception, Prior beliefs, Sensory precision

## Abstract

**Objective:**

Bulimia nervosa (BN) is associated with loss-of-control (LOC) eating episodes that frequently occur in response to negative emotions. According to recent neurocomputational models, this link could be explained by a failure to accurately update beliefs about the body in states of high arousal. Specifically, these interoceptive inference models suggest that under-relying on signals from one’s body about sensory experience (“low sensory precision”) and/or over-relying on previously held beliefs (“excessively precise priors”) lead to inaccurate perception and maladaptive behaviors. We conducted an initial test of these core predictions of the interoceptive inference model in BN using self-report measures.

**Methods:**

We compared women with BN (*n* = 30) and age-, BMI-, and full-scale IQ-matched controls (*n* = 31) on trust in sensory information from the body and two types of beliefs about what can be done to regulate high negative affect. Within the BN group, we tested interrelations among these measures and explored their associations with LOC eating frequency.

**Results:**

Compared with healthy controls, the BN group reported lower levels of trust in sensory information and stronger beliefs that once upset, there is little one can do, apart from eating, to self-regulate. These beliefs were associated with each other and with lower body trust. Beliefs about the uncontrollability of emotion were associated with more frequent subjective binge-eating episodes.

**Conclusions:**

Findings provide initial support for the core predictions of an interoceptive inference account of BN: low trust in sensory information (“sensory precision”) may promote an overreliance on maladaptive “prior beliefs” about the effects of eating on negative emotions, ultimately interfering with accurate updating of beliefs about other strategies that could regulate emotions and maintain LOC eating. Low body trust, strong expectations about emotions, and their neurocomputational underpinnings could be promising combined treatment targets for BN.

**Supplementary Information:**

The online version contains supplementary material available at 10.1186/s40337-024-01010-2.

## Abstract (plain language summary)

Interoception, the brain’s processing of bodily signals, is critical for emotional and behavioral control. Disturbances in interoception may contribute to emotion dysregulation and problematic behaviors across a range of psychiatric disorders, including eating disorders, but the exact mechanisms remain unclear. Recent “interoceptive inference” models of psychopathology propose that dysregulated emotions and maladaptive behaviors persist because, during intense emotional states, individuals under-rely on information from bodily signals and over-rely on pre-existing expectations (“prior beliefs”). In this study, we tested these core predictions among individuals with bulimia nervosa (BN). We compared women with BN and healthy controls on self-reported measures of bodily trust and two types of pre-existing beliefs about responses to negative emotions. We found the first evidence of lower trust in bodily signals in individuals with BN compared to controls. This reduced trust was linked to stronger beliefs that there is little one can do, apart from eating, to regulate emotions. These beliefs, in turn, were associated with more frequent eating episodes characterized by loss of control. Though more research is needed to replicate these results, they provide preliminary support for a model that could explain why individuals with BN are more likely to have uncontrolled eating in the context of strong negative emotions.

**Supplementary Information:**

The online version contains supplementary material available at 10.1186/s40337-024-01010-2.

## Introduction

Recent data suggest that aberrant interoception, or an alteration in one’s ability to detect, interpret, and regulate internal signals related to body states (e.g., hunger, satiety, pain) [1], may be integral to the etiology and maintenance of loss-of-control (LOC) eating and self-induced vomiting in bulimia nervosa (BN) [2–13]. As interoception also supports emotion regulation [14], altered interoception could similarly underpin the affective instability [15] and difficulties with emotion regulation [16–19] that have been consistently documented in BN. However, the specific interoceptive mechanisms that could explain the commonly observed link between momentary increases in negative affect and subsequent binge eating episodes have not been identified [20, 21].

A recent interoceptive inference model explains how altered body signal processing may promote emotion dysregulation and a range of maladaptive behaviors in psychiatric populations [24–29]. Specifically, this model proposes that across psychiatric conditions, the brain appraises afferent bodily signals and associated prediction errors as unreliable proxies of bodily states [22–28]. At the conscious level, this could lead to low levels of trust in sensory information received from the body, preventing accurate perception of changes in bodily state. Computationally, this equates to reduced interoceptive sensory precision estimates. In the interoceptive inference model, these reduced precision estimates lead to perceptions and maladaptive behaviors in response to high arousal that are primarily determined by over-weighted initial expectations or predictions (“hyperprecise prior beliefs”). These hyperprecise priors could be consciously manifested as strongly endorsed and persistent maladaptive beliefs about how high arousal can be managed. Consequently, during bodily state changes (e.g., when negative affect increases), the brain cannot accurately adjust its model of the body, impeding effective self-regulation [29–32].

One prior study found direct partial support for this model in a small, mixed sample of 14 individuals with eating disorders using a Bayesian computational model of perception during an aversive interoceptive perturbation (breath hold). Participants with eating, anxiety, major depressive, and substance use disorders showed reduced heartbeat precision estimates during the interoceptive perturbation [32]. These findings were recently replicated in a larger, but still mixed, sample of 36 individuals with eating disorders [33]. Although task data did not support the model’s assumption of hyperprecise prior beliefs, they suggest that individuals with eating disorders may not trust their bodily signals as reliable sources of information and fail to appropriately adjust sensory precision estimates during state changes introduced by aversive arousal.

Prior self-report research also supports some of the separate components of this model, specifically in BN. Relevant to sensory precision, lower self-reported trust in bodily signals has been linked to more severe eating disorder cognitions, restraint, and binge/purge symptoms [34–36], specifically through emotion dysregulation [37]. However, no studies have directly compared women with BN to matched healthy controls on body trust. Relevant to prior beliefs in states of high negative arousal, several studies have found that individuals with BN are more likely than healthy controls to believe that once they are upset, little can be done self-regulate [18], but they also report stronger beliefs that eating will downregulate their negative affect [38, 39]. These beliefs are highly predictive of LOC eating [40, 41]. However, the interoceptive inference model assumes that low sensory precision can contribute to an overreliance on prior beliefs, and, to our knowledge, the potential associations of low trust in bodily signals with these beliefs have yet to be examined in the same sample of individuals with BN.

Here, we aimed to preliminarily test the core predictions of an interoceptive inference model of BN. We used self-report measures that assess what would be the conscious manifestations of under-weighting of sensory evidence (i.e., reduced trust in sensory information and experience), and over-weighting of prior beliefs (i.e., more strongly endorsing maladaptive beliefs about how sensory perturbation through arousal can be managed), contributing ultimately to maladaptive behavior (i.e., LOC eating). Consistent with the model’s assumption that individuals with BN under-weight signals from the viscera and appraise signals from the body as unreliable, we predicted that women with BN would report lower levels of body trust compared to healthy controls (HC). Consistent with the model’s predictions that under-weighting of body signals could result in an overreliance on strongly held prior beliefs to determine behavior in states of high arousal, we predicted that women with BN would report stronger beliefs that once they are upset, there is little they can do to self-regulate apart from eating, lower body trust would be linked to stronger beliefs, and that these beliefs would be associated with more frequent LOC eating. We also predicted that the beliefs themselves would be correlated, suggesting that strong expectations about eating effects on negative arousal may reinforce strong beliefs that little else could be done to self-regulate and vice versa.

## Methods

### Participants

Participants were right-handed [42] females with (*n* = 30) or without (*n* = 31) *DSM-5* BN [43], aged 18 to 35, weighing between 85 and 120% of the expected weight for their height [44] who participated in a neuroimaging study focused on different forms of cognitive control (NCT02997475; [45]). Women with BN met DSM-5 criteria [43], endorsed purging via self-induced vomiting (though other methods could additionally be endorsed; see Table 1), and if they were taking psychoactive medications, were on a stable dose for at least 4 weeks before study. Women with BN were excluded if they had any comorbid Axis I disorder except for major depression, generalized anxiety disorder, social anxiety disorder, or panic disorder (see Supplement for full inclusion and exclusion criteria). Nine women with BN were receiving behavioral treatment (see Table 1 for additional treatment status information). HC were excluded if they (1) met criteria for the diagnosis of any Axis I psychiatric disorder in their lifetime; (2) had any history of eating disorder behavior, or (3) used psychoactive or other medication known to affect mood or concentration in the last 3 months (see Supplement for full eligibility criteria). Participants were recruited from the UC San Diego Eating Disorders Center for Treatment and Research and the San Diego community. Individuals were screened and characterized using the Mini-International Neuropsychiatric Interview (M.I.N.I.; [46]) and the Structured Clinical Interview for DSM-5 (SCID-5; [47]; see Supplement for further detail), and diagnostic items of the Eating Disorder Examination (EDE; [48]) established BN diagnosis and symptom frequencies. Participant characteristics are presented in Table 1. All participants provided written informed consent, and study procedures were approved by the University of California San Diego’s Human Research Protections Program.

**Table 1:** Sample characteristics

	Healthy controls *N* = 31	Bulimia nervosa *N* = 30		
	M (SD) or *n* (%)	M (SD) or *n* (%)	t, W, or χ2	*p*
Demographics				
Age (years)	22.6 (2.9)	22.6 (3.6)	0.023	0.982
Body mass index (kg/m2)	21.9 (1.8)	21.9 (2.2)	0.120	0.905
WASI-II Full scale IQ score	108.4 (10.0)	107.1 (11.6)	0.453	0.653
Years of education	15.6 (1.7)	15.0 (1.9)	1.243	0.219
Self-reported race	–	–	0.447	0.504
Hispanic	6 (19.4)	3 (10)	–	–
Self-reported ethnicity	–	–	3.972	0.265
White	15 (48.4)	18 (60.0)	–	–
Black/African American	0	0	–	–
Asian	10 (32.3)	11 (36.7)	–	–
Pacific Islander	0	0	–	–
American Indian/Alaska Native	1 (3.23)	0 (0)	–	–
Other	5 (16.1)	1 (3.3)	–	–
Eating disorder symptoms				
Objective bulimic episodes (past 3 months)	–	49.5 (37.6)	–	–
Subjective bulimic episodes (past 3 months)	–	17.00 (25.15)	–	–
Self-induced vomiting (past 3 months)	–	59.8 (48.4)	–	–
Diuretic misuse episodes (past 3 months)	–	4.3 (17.2)	–	–
Laxative misuse episodes (past 3 months)	–	6.5 (25.9)	–	–
Driven and compulsive exercise days (past 3 months)	–	32.6 (28.8)	–	–
Other compensatory behavior days (e.g., chewing and spitting; past 3 months)	–	2.4 (7.3)	–	–
Comorbidities and treatment				
Major depressive disorder	–	8 (26.7)	–	–
Anxiety disorder	–	8 (26.7)	–	–
Generalized anxiety disorder	–	4 (13.3)	–	–
Social anxiety disorder	–	7 (23.3)	–	–
Past anorexia nervosa	–	14 (46.7)	–	–
Hormonal birth control	14 (46.7)	15 (50)	0.01	0.903
Behavioral treatment^a^	–	9 (30)	–	–
Psychotropic medication^b^	–	5 (16.7)	–	–

Note.^a^In the bulimia nervosa group, nine women were receiving behavioral treatment (*n* = 6 outpatient psychotherapy, *n* = 3 partial hospitalization). ^b^In the bulimia nervosa group, five women were taking psychotropic medication at a stable dose for at least 4 weeks (*n* = 1 on escitalopram, *n* = 1 on fluoxetine, *n* = 1 on fluoxetine and gabapentin, *n* = 1 on venlafaxine; *n* = 1 on alprazolam pro re nata (PRN) but abstained from taking this PRN medication in the week prior to study.

### Self-report measures

#### Sensory precision

To measure the conscious manifestation of decreased weighting of sensory evidence, the Multidimensional Assessment of Interoceptive Awareness (MAIA; [49]) Trusting subscale assessed participants’ trust in the reliability of their body signals. The MAIA is a 32-item self-report measure including eight subscales: (1) Noticing, (2) Not-Distracting, (3) Not-Worrying, (4) Attention Regulation, (5) Emotional Awareness, (6) Self-Regulation, (7) Body Listening, and (8) Trusting. Items are rated on a 6-point Likert scale ranging from 0 (never) to 5 (always), with higher scores indicating better IA. We analyzed only the Trusting subscale. Lower scores equate to lower trust. Internal consistency for the subscale in our sample was excellent (*α* = 0.98).

#### Prior beliefs

To measure the conscious manifestation of overreliance on prior beliefs in states of high negative arousal, we assessed general and disorder-specific beliefs about self-regulation strategies in states of high negative affect. The limited access to emotion regulation strategies subscale of the Difficulties in Emotion Regulation Scale (DERS; [50]) assessed general emotion regulation beliefs, specifically, beliefs that there is little one can do to self-regulate once upset. Higher scores indicate stronger beliefs. This subscale showed good internal consistency in our sample (*α* = 0.85). The Eating Helps Manage Negative Affect subscale of the Eating Expectancy Inventory (EEI; [38]) assessed beliefs that eating serves as a successful emotion regulation strategy in high negative affect states (e.g., “When I am feeling anxious or tense, eating helps me relax”). Higher scores indicate stronger beliefs. This subscale showed good internal consistency in our sample (*α* = 0.85).

#### Maladaptive behavior

To examine the model’s assumption that overweighting of prior beliefs contributes ultimately to maladaptive behavior in BN, the Eating Disorder Examination (EDE Version 16.0D; [48]) measured frequencies of BN symptoms. As our disorder-specific prior belief measure only assessed beliefs about the effects of eating (not compensatory behaviors), exploratory analyses focused on maladaptive eating behavior. The EDE assesses objectively large binge-eating episodes (OBEs) and subjectively large binge-eating episodes (SBEs). Both types of episodes are characterized by a sense of loss of control and the perception that the eating episode is large. Because considerable data suggest that the most salient aspect of binge-eating episodes is the sense of LOC over eating [51–53], not objective episode size, we examined the frequencies of both OBEs and SBEs in the past 3 months.

### Statistical analysis

Wilcoxon rank sum tests compared the BN and HC groups on MAIA Body Trust, DERS Limited Access to Emotion Regulation Strategies, and EEI Eating Helps Manage Negative Affect subscales. Within the BN group, robust regressions examined associations among these three subscale scores. In exploratory negative binomial regression models, OBE and SBE episodes were regressed on prior beliefs (DERS Limited Access to Emotion Regulation Strategies and EEI Eating Helps Manage Negative Affect subscales). The false discovery rate (FDR; Benjamini & Hochberg, 1995), controlled for familywise error across the three between-group comparisons, across the three within-group tests, and across the two beliefs for each LOC eating metric.

## Results

**Fig. 1:**
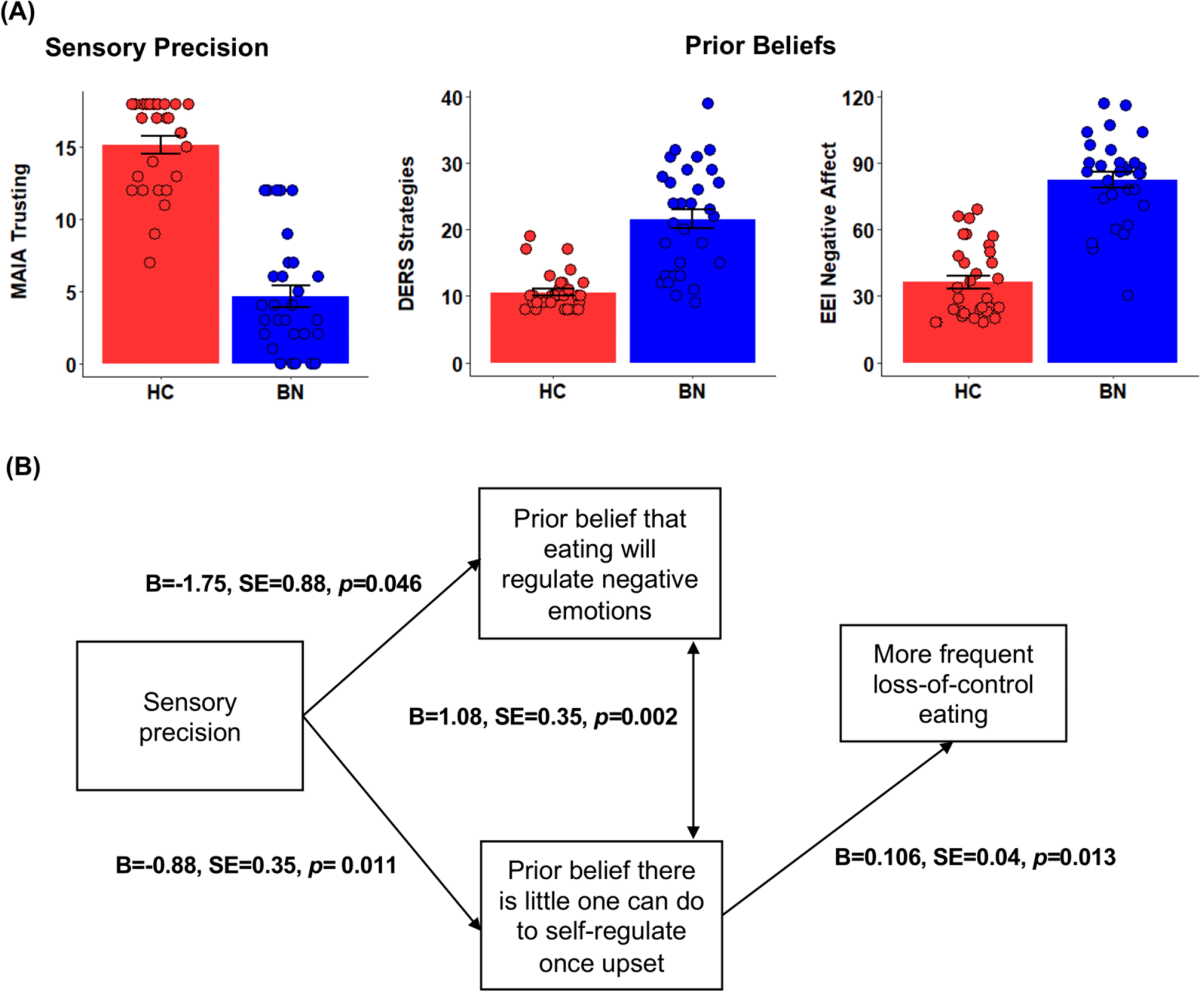
(**A**) Alterations in sensory precision and prior beliefs in BN. Sensory precision was assessed using the body trust subscale of the MAIA, with lower scores indicating lower trust. Beliefs that there is little one can do to self-regulate once upset was assessed using the DERS Strategies subscale, with higher scores indicating stronger beliefs. Beliefs that eating will reduce negative emotions was assessed using the Eating Helps Manage Negative Affect subscale of the EEI, with greater scores indicating stronger beliefs. Groups differed on all three measures (all *p*s_FDR_ < 0.001; error bars indicate SEM). (**B**) Associations among sensory precision, prior beliefs, and symptom severity in BN. All associations were examined in separate analyses with *p* values adjusted for multiple comparisons. All *p*s_FDR_ < 0.05

Women with BN reported significantly lower levels of body trust (*d* = 2.82), stronger beliefs that little can be done to regulate emotions (*d* = 1.83), and stronger beliefs that eating helps regulate negative affect compared to their healthy counterparts (*d* = 2.58, *p*s_FDR_ < 0.001; Fig. 1A).

Figure 1B provides an overview of the separately tested associations among these variables within the BN group. Lower levels of body trust were associated with stronger beliefs that little can be done to help regulate emotions and stronger beliefs that eating will help alleviate negative emotions. These maladaptive beliefs were also correlated (*p*s_FDR_ < 0.05). Stronger beliefs that once upset, little can be done to help regulate emotions predicted more frequent SBEs (*p*_*FDR*_ = 0.026). Beliefs that eating will help regulate negative emotions did not predict OBE or SBE frequency (*p*s *>* 0.60, uncorrected).

## Conclusions

The present proof-of-concept study used self-report measures to test the predictions of a computational model of interoceptive dysfunction in BN. This interoceptive inference model proposes that in altered physiological states (e.g., the high-arousal state linked with negative affect), under-weighted sensory information and over-weighted prior beliefs give rise to misestimations of one’s current bodily state and impede effective self-regulation across psychiatric disorders. We examined self-report measures of these latent neurocomputational processes. Consistent with the model’s predictions that in BN, under-weighting of body signals could result in an overreliance on hyper-precise prior beliefs to determine behavior in states of high arousal, women with BN reported lower levels of body trust that predicted stronger beliefs there is little one can do to self-regulate, apart from eating, once upset, and these stronger prior beliefs about the ineffectiveness of attempts to regulate emotions once upset predicted more frequent subjective binge eating. These preliminary findings, and the neurocomputational framework they support, lay critical groundwork for next-step studies that directly test the influence of reduced sensory precision and hyperprecise prior beliefs on the perceptions, decisions, and behaviors of individuals with BN.

Our case-control results focused on confidence in bodily signals confirm prior task-based findings of reduced interoceptive precision (i.e., under-weighting of body signals) in a large transdiagnostic sample that included a small and mixed subset of individuals with eating disorders [32, 33] and are consistent with previous self-report findings of associations of low body trust and increased symptomatology in eating disorders [34, 36, 37]. Our case-control results focused on beliefs also align with past results indicating that BN is associated with stronger beliefs about the futility of attempts to regulate emotions [18] and stronger expectations that eating will reduce negative emotions [38, 39]. Expanding upon past findings, lower levels of body trust predicted stronger prior beliefs, and these beliefs were positively interrelated in our BN sample. These results provide preliminary support for the notion that low confidence in bodily signals may lead to an overreliance on maladaptive prior beliefs about which responses will be effective in states of high negative affect.

Consistent with the interoceptive inference model assertion that during high arousal, perception, and perhaps behaviors, are primarily driven by over-weighted priors, stronger beliefs that there is little one can do to self-regulate once upset predicted more frequent SBEs. This observed link between beliefs about emotion and SBEs, but not OBEs, may be consistent with data suggesting that SBEs are more strongly related to negative affect [54–56].

Contrary to our hypotheses and some past research [41, 57], beliefs that eating will reduce negative affect were unrelated to OBE or SBE frequencies. We speculate that these beliefs may more closely map onto specific types of binge eating (e.g., planned binge-eating episodes; [58]) or presentations of BN (e.g., the dietary-negative affect subtype; [59] that may have been underrepresented in our sample. Future research stratifying LOC eating episodes based on whether the episodes were planned or intended to reduce negative affect is needed to test this theory. However, taken together, our findings preliminarily suggest that under-weighting of bodily signals may lead to dysregulated eating behaviors primarily determined by increased reliance on certain maladaptive prior beliefs.

These findings could have important clinical implications for improving the efficacy of BN treatment. Although altered neuroendocrine signaling [8, 60, 61] suggests that certain sensory signals (e.g., hunger, fullness) would not effectively guide eating-related decisions in acute stages of illness, targeting low interoceptive precision by promoting trust in other visceral experiences (e.g., via interoceptive exposure) may be effective for BN [62–66]. Over-weighted priors could be targeted with exposures that, informed by inhibitory learning theory, focus on creating experiences that maximally violate strong prior beliefs [67–70]. Future research could test whether these interventions are most effective if delivered in the context of aversive arousal.

Despite the strengths of the current study, including novel investigation of the theoretical constructs underlying an interoceptive model of BN using well-validated measures, study limitations should be noted. First, the reliance on self-report introduces potential biases (e.g., memory recall, meta-cognition) and are not direct measures of the neurocomputational processes outlined in the model. Second, our study was cross-sectional and did not include measures of additional parameters typically included in computational models (e.g., learning rates that determine belief updating speed). Future studies should combine interoceptive prediction tasks with computational modeling and ecological momentary assessments to capture symptomatology in real, longitudinal time and test whether other computational mechanisms of interoceptive processing contribute to BN symptoms. Third, the study included relatively small samples of adult females who were primarily white, and the BN group all endorsed self-induced vomiting. The replication of our findings in larger, more diverse samples is needed.

This is the first study to demonstrate associations among increased body mistrust, maladaptive beliefs, and LOC eating, supporting foundational predictions of an interoceptive inference model of BN. However, longitudinal, task-based, and real-time data are needed to formally test the causal predictions and computational parameters of the model. In addition, computational neuroimaging studies could verify whether altered precision estimates are encoded in the insula and anterior cingulate [22, 71] in BN and examine how dysfunction in the overlapping neural circuits that subserve aversive interoception and emotion regulation may relate to BN symptom severity [8].

### Electronic supplementary material

Below is the link to the electronic supplementary material.

Supplementary Material 1

Supplementary Material 2

Supplementary Material 3

## Data Availability

The datasets used and/or analysed during the current study are available from the corresponding author on reasonable request.

## Abbreviations

*BN*: Bulimia nervosa
*HC*: Healthy control
*DERS*: Difficulties in Emotion Regulation Scale
*DSM-5*: Diagnostic and Statistical Manual of Mental Disorders, 5th Edition
*MAIA*: Multidimensional Assessment of Interoceptive Awareness
*M.I.N.I*: Mini-International Neuropsychiatric Interview
*EEI*: Eating Expectancy Inventory
*OBE*: Objectively large bulimic episode
*SBE*: Subjectively large bulimic episode
*LOC*: Loss-of-control
*SCID-5*: Structured Clinical Interview for DSM-5
*FDR*: False Discovery Rate

## References

[1] Craig AD (2003). Interoception: the sense of the physiological condition of the body. Curr Opin Neurobiol.

[2] Klabunde M, Collado D, Bohon C (2017). An interoceptive model of bulimia nervosa: a neurobiological systematic review. J Psychiatr Res.

[3] Klabunde M (2013). Interoceptive sensitivity deficits in women recovered from bulimia nervosa. Eat Behav.

[4] Lautenbacher S (1991). Pain sensitivity in anorexia nervosa and bulimia nervosa. Biol Psychiatry.

[5] Stein D (2003). Pain perception in recovered bulimia nervosa patients. Int J Eat Disord.

[6] Walsh BT (2003). A disturbance of gastric function in bulimia nervosa. Biol Psychiatry.

[7] Bohon C, Stice E (2011). Reward abnormalities among women with full and subthreshold bulimia nervosa: a functional magnetic resonance imaging study. Int J Eat Disord.

[8] Berner LA (2019). Altered anticipation and processing of aversive interoceptive experience among women remitted from bulimia nervosa. Neuropsychopharmacology.

[9] Oberndorfer TA (2013). Altered Insula response to Sweet taste Processing after Recovery from Anorexia and Bulimia Nervosa. Am J Psychiatry.

[10] Fassino S (2004). Clinical, psychopathological and personality correlates of interoceptive awareness in anorexia nervosa, bulimia nervosa and obesity. Psychopathology.

[11] Lilenfeld LR (2006). Eating disorders and personality: a methodological and empirical review. Clin Psychol Rev.

[12] Pryor T, Wiederman MW (1996). Measurement of nonclinical personality characteristics of women with anorexia nervosa or bulimia nervosa. J Pers Assess.

[13] Taylor GJ (1996). Relationships between alexithymia and psychological characteristics associated with eating disorders. J Psychosom Res.

[14] Craig AD (2008). Interoception and emotion: a neuroanatomical perspective. Handb Emotions.

[15] Santangelo P (2014). Specificity of affective instability in patients with borderline personality disorder compared to posttraumatic stress disorder, bulimia nervosa, and healthy controls. J Abnorm Psychol.

[16] Brockmeyer T (2014). Difficulties in emotion regulation across the spectrum of eating disorders. Compr Psychiatr.

[17] Gilboa-Schechtman E (2006). Emotional processing in eating disorders: specific impairment or general distress related deficiency?. Depress Anxiety.

[18] Harrison A (2010). Emotional functioning in eating disorders: attentional bias, emotion recognition and emotion regulation. Psychol Med.

[19] Svaldi J (2012). Emotion regulation deficits in eating disorders: a marker of eating pathology or general psychopathology?. Psychiatry Res.

[20] Haedt-Matt AA, Keel PK (2011). Revisiting the affect regulation model of binge eating: a meta-analysis of studies using ecological momentary assessment. Psychol Bull.

[21] Lavender JM (2016). Reciprocal associations between negative affect, binge eating, and purging in the natural environment in women with bulimia nervosa. J Abnorm Psychol.

[22] Barrett LF, Simmons WK (2015). Interoceptive predictions in the brain. Nat Rev Neurosci.

[23] Stephan KE (2016). Allostatic self-efficacy: a metacognitive theory of dyshomeostasis-induced fatigue and depression. Front Hum Neurosci.

[24] Smith R (2017). The hierarchical basis of neurovisceral integration. Neurosci Biobehavioral Reviews.

[25] Owens AP (2018). Investigating the relationship between cardiac interoception and autonomic cardiac control using a predictive coding framework. Auton Neurosci.

[26] Paulus MP, Feinstein JS, Khalsa SS (2019). An active inference approach to interoceptive psychopathology. Ann Rev Clin Psychol.

[27] Allen M et al. *In the body’s eye: The computational anatomy of interoceptive inference. bioRxiv. Article 603928*. 2019. 10.1371/journal.pcbi.1010490 PMC9506608 36099315

[28] Gu X, FitzGerald TH, Friston KJ (2019). Modeling subjective belief states in computational psychiatry: interoceptive inference as a candidate framework. Psychopharmacology.

[29] Smith R, Lane RD (2015). The neural basis of one’s own conscious and unconscious emotional states. Neurosci Biobehavioral Reviews.

[30] Critchley HD, Garfinkel SN (2017). Interoception and emotion. Curr Opin Psychol.

[31] Craig AD (2009). How do you feel—now? The anterior insula and human awareness. Nat Rev Neurosci.

[32] Smith R (2020). A bayesian computational model reveals a failure to adapt interoceptive precision estimates across depression, anxiety, eating, and substance use disorders. PLoS Comput Biol.

[33] Lavalley CA et al. Transdiagnostic failure to adapt interoceptive precision estimates across affective, substance use, and eating disorders: a replication study. medRxiv, 2023. 10.1016/j.biopsycho.2024.108825 PMC11416323 38823571

[34] Perry TR (2021). Interoceptive awareness and suicidal ideation in a clinical eating disorder sample: the role of body trust. Behav Ther.

[35] Brown TA (2017). Psychometric evaluation and norms for the Multidimensional Assessment of Interoceptive Awareness (MAIA) in a clinical eating disorders sample. Eur Eat Disorders Rev.

[36] Brown TA (2020). Body mistrust bridges interoceptive awareness and eating disorder symptoms. J Abnorm Psychol.

[37] Brown C, Brown T, Wierenga C (2021). Impact of body trust and emotion regulation on eating disorder severity: a transdiagnostic study. Biol Psychiatry.

[38] Hohlstein LA, Smith GT, Atlas JG (1998). An application of expectancy theory to eating disorders: development and validation of measures of eating and dieting expectancies. Psychol Assess.

[39] Hayaki J (2009). Negative reinforcement eating expectancies, emotion dysregulation, and symptoms of bulimia nervosa. Int J Eat Disord.

[40] Burr EK et al. Emotion regulation difficulties and distress tolerance in predicting loss-of-control eating. Br J Health Psychol, 2022. 10.1111/bjhp.12611 35778877

[41] Pearson CM (2012). A longitudinal transactional risk model for early eating disorder onset. J Abnorm Psychol.

[42] Oldfield RC (1971). The assessment and analysis of handedness: the Edinburgh inventory. Neuropsychologia.

[43] Association AP. *Diagnostic and statistical manual of mental disorders: DSM-5™, 5th ed*. Diagnostic and statistical manual of mental disorders: DSM-5™, 5th ed. 2013, Washington. D.C.: American Psychiatric Association.

[44] Insurance ML. New weight standards for men and women. Stat Bull, 1959. 40(3).

[45] Berner LA et al. Impaired belief updating and devaluation in adult women with bulimia nervosa. Translational Psychiatry, 2023. 13(1). 10.1038/s41398-022-02257-6 PMC9816187 36604416

[46] Sheehan DV (1998). The mini-international neuropsychiatric interview (MINI): the development and validation of a structured diagnostic psychiatric interview for DSM-IV and ICD-10. J Clin Psychiatry.

[47] First MB. *Structured clinical interview for the DSM (SCID)* The encyclopedia of clinical psychology, 2014: pp. 1–6.

[48] Fairburn CG, Cooper Z, O’Connor M. *Eating disorder examination (16.0D)*, in *Cognitive behavior therapy and eating disorders*, C.G. Fairburn, Editor. 2008, Guilford Press: New York.

[49] Mehling WE (2012). The Multidimensional Assessment of Interoceptive Awareness (MAIA). PLoS ONE.

[50] Gratz KL, Roemer L (2004). Multidimensional assessment of emotion regulation and dysregulation: development, factor structure, and initial validation of the difficulties in emotion regulation scale. J Psychopathol Behav Assess.

[51] Goldschmidt AB (2012). Momentary affect surrounding loss of control and overeating in obese adults with and without binge eating disorder. Obesity.

[52] Goldschmidt AB (2017). Are loss of control while eating and overeating valid constructs? A critical review of the literature. Obes Rev.

[53] Derks IP (2022). Subclinical binge eating symptoms in early adolescence and its preceding and concurrent factors: a population-based study. J Eat Disorders.

[54] Brownstone LM, Bardone-Cone AM (2021). Subjective binge eating: a marker of disordered eating and broader psychological distress. Eat Weight Disorders-Studies Anorexia Bulimia Obes.

[55] Brownstone LM (2013). Subjective and objective binge eating in relation to eating disorder symptomatology, negative affect, and personality dimensions. Int J Eat Disord.

[56] Fitzsimmons-Craft EE (2014). Subjective and objective binge eating in relation to eating disorder symptomatology, depressive symptoms, and self‐esteem among treatment‐seeking adolescents with bulimia nervosa. Eur Eat Disorders Rev.

[57] Fischer S, Smith GT (2008). Binge eating, problem drinking, and pathological gambling: linking behavior to shared traits and social learning. Pers Indiv Differ.

[58] Parker MN (2022). Eating expectancies and hedonic hunger among individuals with bulimia-spectrum eating disorders who plan binge‐eating episodes. Int J Eat Disord.

[59] Stice E (2008). Subtyping women with bulimia nervosa along dietary and negative affect dimensions: further evidence of reliability and validity. J Consult Clin Psychol.

[60] Monteleone P, Maj M (2013). Dysfunctions of leptin, ghrelin, BDNF and endocannabinoids in eating disorders: beyond the homeostatic control of food intake. Psychoneuroendocrinology.

[61] Monteleone P, Castaldo E, Maj M (2008). Neuroendocrine dysregulation of food intake in eating disorders. Regul Pept.

[62] Boswell JF (2019). A preliminary naturalistic clinical case series study of the feasibility and impact of interoceptive exposure for eating disorders. Behav Res Ther.

[63] Hildebrandt T, Peyser D, Sysko R (2021). Lessons learned developing and testing family-based interoceptive exposure for adolescents with l ow‐weight eating disorders. Int J Eat Disord.

[64] Zucker N (2017). Acceptance-based interoceptive exposure for young children with functional abdominal pain. Behav Res Ther.

[65] Thompson-Brenner H (2019). Implementation of transdiagnostic treatment for emotional disorders in residential eating disorder programs: a preliminary pre-post evaluation. Psychother Res.

[66] McNally RJ (2016). Can network analysis transform psychopathology?. Behav Res Ther.

[67] Craske MG (2014). Maximizing exposure therapy: an inhibitory learning approach. Behav Res Ther.

[68] Butler RM, et al. Facing eating disorder fears: an Open Trial adapting prolonged exposure to the treatment of eating disorders. Behavior Therapy; 2023. 10.1016/j.beth.2023.07.008 38418045

[69] Schaumberg K (2021). Conceptualizing eating disorder psychopathology using an anxiety disorders framework: evidence and implications for exposure-based clinical research. Clin Psychol Rev.

[70] Reilly EE (2017). Expanding exposure-based interventions for eating disorders. Int J Eat Disord.

[71] Kleckner IR (2017). Evidence for a large-scale brain system supporting allostasis and interoception in humans. Nat Hum Behav.

