# Supplementary material for: Evaluating the predictions of an interoceptive inference model of bulimia nervosa
Source: J Eat Disord. 2024 May 13;12:57. doi: 10.1186/s40337-024-01010-2 (PMC11092174; doi:10.1186/s40337-024-01010-2)
Supplement: Supplementary file 2 — Supplementary Material 2 [file 40337_2024_1010_MOESM2_ESM.docx]

Chester et al.

**SUPPLEMENTARY INFORMATION**

**Supplementary Methods**

**Method**

Women with BN met DSM-5 criteria [1], endorsed purging via self-induced vomiting (though other methods could additionally be endorsed; see Table 1), and if they were taking psychoactive medications, were on a stable dose for at least 4 weeks before study. Women with BN were excluded if they had any comorbid Axis I disorder except for major depression, generalized anxiety disorder, social anxiety disorder, or panic disorder (see Supplement for full inclusion and exclusion criteria). The BN group consisted of 9 women who were receiving behavioral treatment (see Table 1 for additional treatment status information).

**Measures**

The MAIA is a 32-item self-report measure of eight subscales or dimensions of interoceptive awareness: 1) Noticing (e.g., “I notice where in my body I am comfortable”), 2) Not-Distracting (e.g., “I distract myself from sensations of discomfort”), 3) Not-Worrying (e.g., “I can notice an unpleasant sensation without worrying”), 4) Attention Regulation (e.g., “I am able to consciously focus on my body as a whole”), 5) Emotional Awareness (e.g., “When something is wrong in my life, I can feel it in my body”), 6) Self-Regulation (e.g., “When I am caught in my thoughts, I can calm my mind by focusing on my body/breathing”), 7) Body Listening (e.g., “I listen to my body to inform me about what to do”), and 8) Trusting (e.g., “I trust my body sensations”). Items are rated on a 6-point Likert scale ranging from 0 (never) to 5 (always), with higher scores indicating higher levels of interoceptive awareness. The MAIA subscales have demonstrated acceptable psychometric properties in individuals with eating disorders [2] and other populations [3, 4].

The Eating Expectancy Inventory (EEI; [5]) is a 34-item self-report measure that assesses cognitive expectancies for reward and relief from negative affect after eating and is comprised of five subscales. The EEI has demonstrated good internal consistency and construct validity across eating disorder populations and both psychiatric and non-psychiatric controls [38].

**Full Inclusion and Exclusion Criteria**

Participants included right-handed [6] females aged 18 to 35 and between 85 and 120% of the expected weight for their height based on the Metropolitan Life Insurance tables [7]. Women in the bulimia nervosa (BN) group met *DSM-5* criteria (at least one objective bulimic episode and compensatory behavior per week for the past three months) [1], endorsed purging via self-induced vomiting (though other methods could additionally be endorsed), and were on a stable dose of all psychoactive medications for at least 4 weeks before study.

Healthy controls were excluded if they 1) met criteria for the diagnosis of any Axis I psychiatric disorder at any point in their lifetime; 2) endorsed any history of binge eating (including any history of an experience of “loss of control” over eating) or self-induced vomiting, laxative or diuretic misuse, or 3) had used psychoactive or other medications known to affect mood or concentration in the last 3 months.

Women with BN were excluded if they had 1) any comorbid Axis I disorder except for major depression, generalized anxiety disorder, social anxiety disorder, or panic disorder, 2) a primary diagnosis of any of these comorbidities; 3) any psychopathology that might interfere with their ability to participate in the study (e.g., requiring inpatient hospitalization).

Universal exclusion criteria across both groups were: 1) current significant medical illness; 2) substance use disorder in the past 6 months; 4) pregnancy or planned pregnancy during the study period, or lactation; 5) current or past neurological disorder or organic brain syndrome or dementia; 6) history of a seizure or head trauma with loss of consciousness; 6) Full Scale IQ under 75.

Participants were screened for exclusionary psychiatric disorders using the Mini-International Neuropsychiatric Interview [M.I.N.I.; 8], and, for disorders not included in the M.I.N.I., the Structured Clinical Interview for *DSM-5* [SCID-5; 9]. Included disorders (eating disorders, major depression, anxiety disorders, substance use disorder prior to 6 months ago) were more comprehensively assessed using the SCID-5. A modified structured interview used in neuroimaging studies of individuals with past eating disorders [10, 11] using diagnostic items of the Eating Disorder Examination (EDE; [12]) established BN diagnosis and symptom frequencies. The two-subtest Wechsler Abbreviated Scale of Intelligence (WASI-II; [13]) estimated general intellectual functioning (FSIQ).

**References**

1. American Psychiatric Association, *Diagnostic and Statistical Manual of Mental Disorders: Fifth Edition (DSM-5)*. 2013, Washington. D.C.: American Psychiatric Association.

2. Brown, T.A., et al., *Psychometric evaluation and norms for the Multidimensional Assessment of Interoceptive Awareness (MAIA) in a clinical eating disorders sample.* European Eating Disorders Review, 2017. **25**(5): p. 411-416.

3. Machorrinho, J., et al., *Multidimensional assessment of interoceptive awareness: Psychometric properties of the Portuguese version.* Perceptual and motor skills, 2019. **126**(1): p. 87-105.

4. Mehling, W.E., et al., *The Multidimensional Assessment of Interoceptive Awareness (MAIA).* PLoS One, 2012. **7**(11): p. e48230.

5. Hohlstein, L.A., G.T. Smith, and J.G. Atlas, *An application of expectancy theory to eating disorders: Development and validation of measures of eating and dieting expectancies.* Psychological Assessment, 1998. **10**(1): p. 49.

6. Oldfield, R.C., *The assessment and analysis of handedness: the Edinburgh inventory.* Neuropsychologia, 1971. **9**(1): p. 97-113.

7. Metropolitan Life Insurance Company, *New weight standards for men and women*, in *Stat Bull Metrop Insur Co*. 1959. p. 1-11.

8. Sheehan, D.V., et al., *The Mini-International Neuropsychiatric Interview (M.I.N.I.): the development and validation of a structured diagnostic psychiatric interview for DSM-IV and ICD-10.* The Journal of Clinical Psychiatry, 1998. **59** (Suppl 20): p. 22-33.

9. First, M., et al., *User’s Guide for the Structured Clinical Interview for DSM-5 Disorders, Research Version (SCID-5-RV). Arlington, VA, American Psychiatric Association.* 2015.

10. Wierenga, C.E., et al., *Increased anticipatory brain response to pleasant touch in women remitted from bulimia nervosa.* Translational Psychiatry, 2020. **10**(1): p. 236.

11. Berner, L.A., et al., *Altered anticipation and processing of aversive interoceptive experience among women remitted from bulimia nervosa.* Neuropsychopharmacology, 2019. **44**(7): p. 1265-1273.

12. Fairburn, C.G., Z. Cooper, and M. O’Connor, *Eating disorder examination (16.0D)*, in *Cognitive behavior therapy and eating disorders*, C.G. Fairburn, Editor. 2008, Guilford Press: New York.

13. Wechsler, D., *Wechsler Abbreviated Scale of Iintelligence–Second Edition [Database record].* APA PsycTests. <https://doi>. org/10.1037/t15171-000, 2011.
